# The Therapeutics of Music

**Published:** 1891-10-17

**Authors:** 


### Passing Topics.

### THE THERAPEUTICS OF MUSIC.

The interesting pamphlet published some years ago under this title by Dr. G. Herbert Lilley, Medical Officer of H.M. Convict Prison, Portland, has recently attracted an attention which it seems to have been denied upon its first appearance.

Possibly the suggestion—not quite novel either—that music could exert a real curative power upon disease has needed time to take root, or it may be that the advances in the diagnosis and treatment of nervous maladies have brought the faculty, to a point at which they are enabled better to estimate the subtle effect of music upon those delicate nerve centres whence proceed the emotions which affect in so powerful a degree the whole human organism. At any rate, the faculty, we notice, is not prepared to treat Dr. Lilley's essay as altogether fanciful, and when we reflect for a moment upon the suffering caused to many people, not in other respects hyper-sensitive, by the infliction of discordant sounds, it seems only natural that pleasing and harmonious strains may exert the soothing influence which is all important to the sick. If music do no more than induce a feeling of repose a great deal must be gained, and the fact that in certain cases even the most melodious notes would cause irritation proves nothing more than that the nervous system may become as abnormally irritable, as the stomach does when it refuses to retain food, no matter how nutritious.

Dr. Lilley's assertion that "with very few exceptions all insane persons like music" offers a fine occasion for critical remark and not a little satire, while in regard to those efforts now so general in hospitals and asylums to provide musical entertainments for the patients, we fear it is not altogether encouraging to be told that if music is to be of any value therapeutically it must not only be well chosen and well executed, but it must be "scrupulously considered in regard to individual natures." The notion of adjusting instrument, air, and performer to the particular condition of each separate sufferer would appear to open up a vista of difficulties too tremendous to be lightly faced. Care would need to be taken to prevent any portion of the potion allotted to one patient from overflowing, as it were, into the ears of another, and unless the majority were able to absorb, unharmed, a mixture of tunes it would be impossible in a large establishment to find time to allot to every individual his daily dose.

But although the subject has its ludicrous aspect it is by no means wholly funny, and even were its promises without the high sanction of professional opinion, we should not be sceptical. Some time ago we advocated in this column the establishment of hospital choirs, and although we prefer the notion that each hospital should make musical provision of its own, we not the less welcome the establishment of the St. Cecilia's Guild, about which we may have something to say later on. Hospital choirs and St. Cecilia's Guild, however, are concerned to promote the common weal, and while, as Dr. Lilley argues, music has been allowed in all ages to possess healing properties, it is left to the science of the present day to show, if it can, how these can be utilised in the interests of individual sufferers. Agricola.

